# Supplementary material for: Engagement in research capability-building: Impact on healthcare workforce attraction and retention in rural and remote Australia – A scoping review protocol
Source: PLoS One. 2026 Jan 28;21(1):e0340289. doi: 10.1371/journal.pone.0340289 (PMC12851435; doi:10.1371/journal.pone.0340289)
Supplement: S1 Table — (DOCX) [file pone.0340289.s001.docx]

**S1 Table**: Search strategy for a scoping review of engagement in research capability-building and its impact on healthcare workforce attraction and retention in rural and remote Australia.

| **Concepts** | Concept 1 | Concept 2 | Concept 3 | Concept 4 |
| --- | --- | --- | --- | --- |
|  | **Healthcare workforce** | **Engagement in research and capability budling initiatives/programs/strategies** | **Attraction and retention** | **Rural and Remote Australia settings** |
| **Subject headings** | | | | |
| Medline | exp Health Personnel/ OR  exp Workforce/ OR exp Health Workforce/ OR exp Physicians/ OR exp Allied Health Personnel/ OR exp Allied Health Occupations/ OR  exp Nurses/ OR exp Health Occupations/ | exp Capacity Building/ OR exp Mentoring/ OR exp Mentors/ OR exp Research/ OR exp Health Services Research/ OR  exp Program Evaluation/ OR exp Evidence-Based Practice/ OR exp Evidence-Based Medicine/ OR exp Translational Research, Biomedical/ OR exp Nursing Research/ | Job Satisfaction/ OR Personnel Management/ OR Personnel Turnover/ OR exp Organizational Innovation/ OR Organizational Culture/ OR exp Staff Development/ OR exp Burnout, Professional/ OR exp Job Security/ OR exp Efficiency, Organizational/ OR exp Workplace/ OR exp employment/ | exp Australia/ OR exp Australian capital territory/ OR exp new south Wales/ OR exp northern territory/ OR exp Queensland/ OR exp south Australia/ OR exp Tasmania/ OR exp Victoria/ OR exp western Australia/  ---------------------------------------------------  exp Rural Health/ OR  exp Rural Health Services/ OR  exp Primary Health Care/ OR exp Vulnerable Populations/ OR exp Medically Underserved Area/ OR exp Healthcare Disparities/ |
| Global Health/ Ovid | exp health care workers/ OR exp medical auxiliaries/ OR exp physicians/ OR exp hospital personnel/ | exp research support/ OR exp interdisciplinary research/ OR exp "implementation of research"/ OR exp nutrition research/ OR exp research/ OR exp operations research/ OR exp medical research/ | exp work satisfaction/ OR exp burnout/ OR  exp working conditions/ OR exp employment/ | exp Australia/ OR Australian capital territory/ OR new south Wales/ OR northern territory/ OR Queensland/ OR south Australia/ OR Tasmania/ OR Victoria/ OR western Australia/  ---------------------------------------------------  exp rural areas/ OR exp rural communities/ OR exp rural health/ OR exp disadvantaged/ OR  exp less favoured areas/ |
| CINAHL Ultimate/ EBSCO | (MH "Health Personnel+") OR (MH "Rural Health Personnel+") OR (MH "Health Occupations+") OR (MH "Physicians+") OR (MH "Nurses+") | (MH "Research+") OR (MH "Research Support+") OR (MH "Action Research") OR (MH "Mentorship") OR (MH "Professional Practice, Research-Based+") OR (MH "Medical Practice, Research-Based") OR (MH "Nursing Practice, Research-Based") OR (MH "Physical Therapy Practice, Research-Based") OR (MH "Occupational Therapy Practice, Research-Based") | (MH "Personnel Retention") OR (MH "Stability+") OR (MH "Work Engagement+") OR (MH "Job Satisfaction+") OR (MH "Burnout, Professional+") OR (MH "Employment+") | (MH "Australia+") OR (MH "Australian Capital Territory") OR (MH "New South Wales") OR (MH "Northern Territory") OR (MH "Queensland") OR (MH "South Australia") OR (MH "Tasmania") OR (MH "Victoria") OR (MH "Western Australia")  ---------------------------------------------------  (MH "Hospitals, Rural") OR (MH "Rural Health") OR (MH "Rural Health Services") OR (MH "Services for Australian Rural and Remote Allied Health") OR (MH "Remote Area Nurses") OR (MH "Remote Area Nursing") OR (MH "Medically Underserved Area") OR (MH "Rural Areas") OR (MH "Medically Underserved") OR (MH "Personnel Shortage+") OR (MH "Nursing Shortage") OR (MH "Medication Shortage") OR (MH "Catchment Area (Health)") |
