# Supplementary material for: Engagement in research capability-building: Impact on healthcare workforce attraction and retention in rural and remote Australia – A scoping review protocol
Source: PLoS One. 2026 Jan 28;21(1):e0340289. doi: 10.1371/journal.pone.0340289 (PMC12851435; doi:10.1371/journal.pone.0340289)
Supplement: Attachment — Submitted filename: Response to reviewers_Haile et al.docx [file pone.0340289.s003.docx]

**November 24, 2025**

Muhammad Shahzad Aslam, Ph.D.,M.Phil., Pharm-D

Academic Editor

PLOS ONE

**Re: Response to reviewers – PONE-D-25-29995**

Dear Editor,

We sincerely appreciate the opportunity to revise our manuscript, *Engagement in research capability-building: Impact on healthcare workforce attraction and retention in rural and remote Australia – A scoping review protocol*, for further consideration for publication in *PLOS ONE*. We are grateful for the thoughtful and constructive comments provided by the reviewers, which will strengthen our actual scoping review work.

We have carefully addressed all comments and incorporated the suggested changes into the revised protocol. A detailed point-by-point response outlining the revisions is provided in the following pages.

Thank you for your time and consideration. We hope the revised manuscript meets the journal’s standards and is acceptable for publication.

Kind regards,

**Authors – PONE-D-25-29995**

**Manuscript Title**: Engagement in research capability-building: Impact on healthcare workforce attraction and retention in rural and remote Australia – A scoping review protocol

**Manuscript ID:** PONE-D-25-29995

**Response to Editor’s and Reviewers’ comments**

Editor’s comments

**General comments**

Both reviewers recognise the importance of exploring how research engagement and capability-building may support recruitment and retention of the rural and regional health workforce. Nevertheless, they highlight that the manuscript requires clearer conceptual framing, justification of scope, and refinement of methods to ensure transparency and reproducibility.

**Authors:** Dear Editor, we sincerely appreciate the time and effort devoted to reviewing our manuscript and for the thoughtful, constructive feedback provided. Your comments have been invaluable in strengthening our scoping review, which aims to map and synthesise the evidence on research capability building and its relationship to health workforce outcomes. We have carefully considered all suggestions and incorporated the recommended revisions. A detailed, point-by-point response and clarification to each comment is provided below.

I concur with the reviewers that the scoping review approach is appropriate for this exploratory topic and should be explicitly and consistently presented throughout the manuscript. Below, I summarise the required and recommended revisions.

Comment 1: Clarify and align the review type.

**Response:** Thank you for this suggestion. Our study is a scoping review, aiming to map the available evidence, identify the enablers and barriers of implementation, and identify best strategies for improving research capability-building and its influence on health workforce attraction and retention in Australia. We have clarified this and ensured that the terminology is consistently aligned throughout the manuscript.

Comment 2: Justify the scope

**Response**: Dear Editor, thank you for your comment. Australia faces critical health workforce shortages, particularly in rural and remote areas. To address this, Australian health departments, research institutes, and academic institutions have invested in research capability-building (RCB) initiatives to strengthen skills, retention, and performance among rural health professionals. While similar initiatives exist internationally, evidence on their impact within the Australian context remains fragmented. This scoping review focuses specifically on Australia to map current evidence, identify national-level barriers and enablers, and generate insights that can guide culturally and contextually tailored strategies, which ultimately aims to enhance the effectiveness of RCB programs on workforce outcomes and healthcare delivery at individual, team, organizational, and system levels in rural and remote Australia.

Comment 3: Define key terms and maintain consistency

**Response:** Thank you for highlighting this. We have carefully revised the manuscript to define all key terms, ensure consistent use of abbreviations, and maintain uniform terminology throughout the document.

Comment 4: Strengthen the rationale and theory of change

**Response**: We thank you for your suggestion. We have strengthened the manuscript by clarifying the rationale for this scoping review, highlighting gaps in evidence on RCB and its impact on health workforce outcomes in rural and remote Australia. We will also demonstrate how RCB contributes at the individual, organizational/team, and system levels using a socio-ecological model, and also, we will illustrate the mechanisms through which these initiatives can influence workforce outcomes including attraction, retention, and stability. Our revision can be found in the background section on pages 3 and 4, lines 69 to 85 and method section, on the analysis (page 10, presented in an outlined Figure 1).

Comment 5: Integrate existing evidence and program outcomes.

**Response**: Thank you for your comment. We will incorporate existing evidence on RCB initiatives and their reported outcomes in rural and remote Australia. This will include both published studies and relevant program reports, highlighting the effects of RCB on workforce outcomes including skills, retention, attraction, career growth, and performance. By integrating these findings, we aim to provide a comprehensive overview of what is currently known, identify gaps in evidence, and inform best practices and future program design to enhance the impact of RCB on health workforce outcomes.

Comment 6: Provide detailed search strategy and feasibility statement.

**Response**: Thank you for your suggestion. We have developed a thorough search strategy covering key databases such as Medline, Global Health, CINAHL, Scopus, and ProQuest Central to identify all relevant studies on research capability-building and its effect on health workforce attraction and retention in rural and remote Australia. To capture grey literature, we will also search Google Scholar, Google, and resources from relevant organizations and rural health research institutions. Our searches indicate that the strategy is feasible and able to retrieve a sufficient number of relevant studies. This approach ensures the review is methodologically robust and practical within the planned timeframe, providing a strong basis for systematically mapping the evidence.

Comment 7: Minor textual corrections.

Response: Thank you for your suggestions. We have carefully revised the manuscript to address grammatical issues, improve clarity, and ensure consistency throughout the text. A thorough proofreading has been conducted to enhance readability and overall presentation.

**Specific comments**

Comment 1: The manuscript and title indicate a scoping review protocol (appropriate given the likely heterogeneity and exploratory state of the evidence). Ensure all instances in the text, abstract, and methods consistently describe a scoping (not systematic) review and that the rationale follows PRISMA-ScR logic (i.e., mapping concepts, clarifying definitions, identifying gaps, not pooling/comparing effects).

**Response:** Then you for your comments. Our work is a scoping review, and we revised thoroughly and adhere to the PRISM-ScR logic to properly map concepts, clarifying definitions, identify evidence gaps and best strategies. Thus, we provided the details of our scoping review methods which we outlined to follow the PRISMA-ScR logic.

Comment 2: Expand to include OECD/high-income rural/remote contexts (with Australia as a prespecified subgroup) to enhance yield and generalisability; or Retain Australia-only but explicitly justify (policy relevance, programmatic uniqueness, feasibility) and discuss implications for transferability.

Response: Dear Editor, thank you for this valuable suggestion. Our current scoping review focuses exclusively on Australia to capture culturally and contextually specific strategies for strengthening the rural and remote health workforce. Australia’s unique geographic, demographic, and health system characteristics, alongside ongoing national research capability-building initiatives, make this focus particularly relevant for informing local policy and program design. While we recognize the potential value of including other high-income countries, we plan to explore broader international evidence in a future systematic review. Nonetheless, the insights generated from the Australian context are likely to be transferable to settings with comparable rural health challenges and system structures, such as Canada, supporting broader applicability of our findings. The rationale for this Australia-specific focus is detailed in the Background section (page 4, lines 86 to 92).

Comment 3: Define your concept of interest (research engagement/capability-building)

Response: We defined our concept, research capacity-building, on page 4 and provide as follows:

“RCB involves strengthening the skills, systems, resources, and organisational structures that enable individuals and institutions to generate, interpret, and apply research to improve practice and decision-making(Cooke, 2005).”

Comment 4: State how you will contextualise findings alongside other known strategies in the discussion (e.g., mapping co-interventions, noting where research is embedded within larger retention packages).

**Response**: Thank you for this suggestion. In the Discussion, we will position Australian RCB initiatives alongside other workforce retention strategies in rural and remote settings, such as mentoring, financial incentives, and professional development programs. We will map how RCB programs interact with broader retention efforts, highlighting co-interventions, potential synergies, and gaps. This approach will allow us to identify best practices and provide practical, evidence-informed recommendations to strengthen workforce attraction, retention, and sustainability across individual, team, organizational, and system levels in rural and remote Australia. The revised text appears in the Discussion section (page 12, lines 257 to 264) as follows:

“Our scoping review will also position RCB within the broader suite of interventions designed to strengthen the rural and remote health workforce. We will examine how capability-building initiatives intersect with complementary strategies, such as professional development programs, mentoring and supervision schemes, financial incentives, and organisational retention efforts. By identifying co-interventions, potential synergies, and areas of duplication or missed opportunity, the review will highlight best practices and generate evidence-informed recommendations to enhance attraction, retention, and workforce sustainability at individual, team, organisational, and system levels.”

Comment 5**:** Use one set of geographic terms throughout. If Australia-specific classifications are needed, define at first use.

**Response**: Dear Editor, thank you for your suggestion. We have revised the manuscript to use a consistent set of geographic terms throughout, with Australia as the primary focus. Any Australia-specific classifications are clearly defined at their first mention to ensure clarity and consistency for readers.

Comment 6: Specify Population–Concept–Context

**Response**: We specify the Population-Concept-Context (PCC) approach, and our revision founds on page 6, lines 133 to 140, and reads now as:

“We used the population, concepts, and context (PCC) approach to formulate the review questions. Accordingly, the participants population will include all health professionals including the four professional categories: nurses and midwives, medical practitioners, dental practitioners, and allied health according to the AIHW Health workforce report. The concept (phenomena of interest) is engagement in research or capability building and its effect on workforce attraction, retention and / or stability. Lastly, the context includes remote and rural areas of Australia which could consists of remote areas, disadvantaged areas, underserved areas, and or far to reach areas.”

Comment 7: Provide a transparent draft search string (per database) and list of key terms/synonyms

**Response**: Thank you for your comment. We have developed clear and transparent search strings for each database, incorporating key terms, synonyms, and subject headings aligned with four core concepts: (1) healthcare workforce, (2) research and capacity-building initiatives, (3) workforce outcomes including attraction and retention, and (4) rural and remote Australian settings. A sample search strategy for each database is presented **in Table 1**, and the full detailed strategies are provided as a supporting file to ensure reproducibility and allow readers to assess our methodology. This approach maximizes transparency and rigor in the review process.

**Table 1.** Sample search strategy for major search databases

| Database | Concept 1 | Concept 2 | Concept 3 | Concept 4 |
| --- | --- | --- | --- | --- |
|  | **Healthcare workforce** | **Research and research capability budling initiatives/programs/strategies** | **Health workforce outcomes (Attraction and retention)** | **Rural and remote settings and Australia** |
| Medline (Ovid) | exp Health Personnel/ OR  exp Workforce/ OR exp Health Workforce/ OR exp Physicians/ OR exp Allied Health Personnel/ OR exp Allied Health Occupations/ OR  exp Nurses/ OR exp Health Occupations/ | exp Capacity Building/ OR exp Mentoring/ OR exp Mentors/ OR exp Research/ OR exp Health Services Research/ OR  exp Program Evaluation/ OR exp Evidence-Based Practice/ OR exp Evidence-Based Medicine/ OR exp Translational Research, Biomedical/ OR exp Nursing Research/ | Job Satisfaction/ OR Personnel Management/ OR Personnel Turnover/ OR exp Organizational Innovation/ OR Organizational Culture/ OR exp Staff Development/ OR exp Burnout, Professional/ OR exp Job Security/ OR exp Efficiency, Organizational/ OR exp Workplace/ OR exp employment/ | exp Australia/ OR exp Australian capital territory/ OR exp new south Wales/ OR exp northern territory/ OR exp Queensland/ OR exp south Australia/ OR exp Tasmania/ OR exp Victoria/ OR exp western Australia/  ----------------------------------------------------------------------------------  exp Rural Health/ OR  exp Rural Health Services/ OR  exp Primary Health Care/ OR exp Vulnerable Populations/ OR exp Medically Underserved Area/ OR exp Healthcare Disparities/ |
| Global Health (Ovid) | exp health care workers/ OR exp medical auxiliaries/ OR exp physicians/ OR exp hospital personnel/ | exp research support/ OR exp interdisciplinary research/ OR exp "implementation of research"/ OR exp nutrition research/ OR exp research/ OR exp operations research/ OR exp medical research/ | exp work satisfaction/ OR exp burnout/ OR  exp working conditions/ OR exp employment/ | exp Australia/ OR Australian capital territory/ OR new south Wales/ OR northern territory/ OR Queensland/ OR south Australia/ OR Tasmania/ OR Victoria/ OR western Australia/  ----------------------------------------------------------------------------------  exp rural areas/ OR exp rural communities/ OR exp rural health/ OR exp disadvantaged/ OR  exp less favoured areas/ |
| CINAHL Ultimate (EBSCO) | (MH "Health Personnel+") OR (MH "Rural Health Personnel+") OR (MH "Health Occupations+") OR (MH "Physicians+") OR (MH "Nurses+") | (MH "Research+") OR (MH "Research Support+") OR (MH "Action Research") OR (MH "Mentorship") OR (MH "Professional Practice, Research-Based+") OR (MH "Medical Practice, Research-Based") OR (MH "Nursing Practice, Research-Based") OR (MH "Physical Therapy Practice, Research-Based") OR (MH "Occupational Therapy Practice, Research-Based") | (MH "Personnel Retention") OR (MH "Stability+") OR (MH "Work Engagement+") OR (MH "Job Satisfaction+") OR (MH "Burnout, Professional+") OR (MH "Employment+") | (MH "Australia+") OR (MH "Australian Capital Territory") OR (MH "New South Wales") OR (MH "Northern Territory") OR (MH "Queensland") OR (MH "South Australia") OR (MH "Tasmania") OR (MH "Victoria") OR (MH "Western Australia")  ----------------------------------------------------------------------------------  (MH "Hospitals, Rural") OR (MH "Rural Health") OR (MH "Rural Health Services") OR (MH "Services for Australian Rural and Remote Allied Health") OR (MH "Remote Area Nurses") OR (MH "Remote Area Nursing") OR (MH "Medically Underserved Area") OR (MH "Rural Areas") OR (MH "Medically Underserved") OR (MH "Personnel Shortage+") OR (MH "Nursing Shortage") OR (MH "Medication Shortage") OR (MH "Catchment Area (Health)") |

Comment 8: Elaborate and explain in detail Study selection & calibration

**Response**: Thank you for this suggestion. We have expanded and clarified our study selection and calibration procedures as follows:

Initially, all records from database and grey literature searches will be imported into Covidence™, and duplicates will be removed automatically and manually verified. Then we’ll follow subsequent steps based on the PRISMA-ScR framework.

First, before screening, we will undertake a calibration exercise in which two reviewers (TGH and ME) independently screen a random sample of 25 to 50 titles and abstracts using the draft eligibility criteria. Discrepancies will be discussed to refine the criteria and screening instructions.

Second, both reviewers will independently screen all titles and abstracts in Covidence™ against the inclusion criteria, coding each record as “include,” “exclude,” or “uncertain.” Conflicts will be resolved through discussion, with a third reviewer available for arbitration.

Third, full texts will be retrieved for all records marked as “include” or “uncertain,” and the two reviewers will independently assess eligibility. Reasons for exclusion will be documented using predefined categories. Any disagreements will be resolved by discussion.

Fourth, backward and forward citation tracking will be conducted by one reviewer and cross-checked by the second, with newly identified records undergoing the same screening process. The entire study selection process will be documented and presented in a PRISMA-ScR flow diagram, with details of records screened, excluded (with reasons), and included reported in an appendix.

Finally, data extraction will follow a structured extraction tool developed in Microsoft Excel™. The tool will be piloted by both reviewers on a sample of five included studies and refined as needed. Following piloting, both reviewers will independently extract data and meet regularly to ensure accuracy, completeness, and consistency.

Comment 9: Describe narrative mapping and tabulation, grouping by programme type, profession, and context; depict the programme theory as a diagram; identify evidence gaps and future research needs. No meta-analysis is expected.

**Response**: Thank you for this insightful suggestion. We have now expanded the analysis plan to provide a clearer description of how narrative mapping, tabulation, and programme-level grouping will be undertaken. Our analysis will begin with a structured descriptive summary of all included studies, presented through narrative mapping and comprehensive tables that categorise the evidence by programme type, health profession, setting, and broader implementation context. We will then synthesise findings using the deductive socioecological model and the inductively developed frameworks on RCB enablers, barriers, and workforce outcome pathways.

Furthermore, to enhance conceptual clarity, we will develop a programme theory diagram illustrating how RCB activities operate across individual, team, organisational, and system levels, and how these mechanisms contribute to workforce outcomes. As part of the synthesis, we will identify areas where evidence is limited or inconsistent and highlight key gaps that warrant future research.

**Reviewers’ comments**

**Reviewer #1,** Kelley Arredondo

**Authors:** Dear reviewer, thank you for your insightful and constructive feedback. Your suggestions have been invaluable in enhancing the clarity, scope, and methodological rigor of our protocol. We have carefully considered and incorporated your comments and have provided further point-by-point clarifications in the following pages.

Comment 1: Workforce attraction: I **suggest workforce recruitment** unless workforce attraction is the necessary specific term.

**Response**: Thank you for this helpful suggestion. While recruitment is a common human-resources term, our review focuses on broader workforce outcomes rather than operational hiring processes. We have deliberately used the term workforce attraction to capture the factors that encourage health professionals to consider working in rural and remote areas, essentially, what generates initial interest in these positions. In contrast, workforce retention addresses the mechanisms that support professionals to remain in these settings over time, including job satisfaction, professional development, mentorship, and supportive organisational and system-level factors. This distinction ensures that our review comprehensively examines the continuum of workforce outcomes, encompassing attraction, retention, stability, and related measures, rather than limiting the scope to recruitment alone.

Comment 2: Research capability building sounds a bit clunky, if this is not an already used term then I suggest research skill development. If it is the accepted term, please ignore.

**Response**: Dear reviewer, thank you for this suggestion. We have chosen to retain the term RCB because it is well-established in the literature to describe structured initiatives that enhance health professionals’ ability to engage in research, beyond simply acquiring discrete skills. RCB encompasses the development of knowledge, practical skills, confidence, and organisational support necessary to conduct research effectively. This usage is supported by prior studies in health workforce and implementation research(Cooke, 2005; Dako-Gyeke et al., 2020; Flenady et al., 2022; Matus et al., 2018), which consistently describe RCB as a comprehensive approach to strengthening research engagement and capacity. Retaining this term ensures conceptual clarity and aligns our work with established frameworks in the field.

Comment 3: Spell out acronyms the first time you use them like FTE in the first paragraph.

**Response**: Thank you for this suggestion. We have revised the manuscript to ensure that all abbreviations and acronyms are spelled out at first use and are consistently used throughout the document thereafter.

Comment 4: Please use consistent terminology. Currently there is: remote and very remote, rural and regional communities, and rural and remote. If these specific terms are used for a reason, please define.

**Response**: Dear reviewer, thank you for your observation. We have revised the manuscript to consistently use the terms rural and remote settings throughout. In the development of our search strategy, however, we included related terms such as very remote and regional to reflect the terminology used in the literature and to ensure comprehensive capture of relevant studies.

Comment 5: In the intro, line 63 please use a phrase like “for example” when noting that research shows that medical grads are less willing to consider rural practice since the paper is inclusive to all clinicians, not just physicians.

**Response**: Thank you, revised and included the phrase “for example”.

Comment 6: Are there reported outcomes for individuals who have participated in RRCBP or STaRR in terms of career development, satisfaction of participant’s career, rural retention of participants, etc.?

**Response**: Dear reviewer, thank you for raising this important point. There is empirical evidence from Australian research capacity‑building programmes that demonstrates positive outcomes in career development, satisfaction, and rural retention among health professionals participated/engaged in RCB programs.

- A qualitative follow-up of graduates from the Rural Research Capacity Building Programme (RRCBP) found that participants reported enhanced research skills, confidence, and employability, as well as embedded research capacity in their teams and organisations(Schmidt et al., 2022). Several graduates noted that participation motivated them to remain in rural roles longer by providing professional growth and a sense of purpose.
- The STaRR (Supporting Translation of Research in Rural and Regional Settings) program has enabled emerging researchers to lead or contribute to practice-based research in rural health services. Early evaluations indicate that the mentoring and workshop components foster research networks, strengthen research capability, build social capital, and support research translation(King et al., 2025).
- Further, embedding research into the organisational structure of smaller rural hospitals has been shown to strengthen research culture and capacity. Staff involved in such programs reported perceived benefits for their career development and expressed increased motivation to remain in rural health settings(Alston et al., 2025; King et al., 2025).

These findings support that RCB initiatives, including RRCBP, STaRR, and embedded hospital research programs, contribute to improved workforce career development, job satisfaction, and retention, highlighting their potential to strengthen the rural health workforce in Australia.

Comment 7: Please provide more evidence that research capacity impacts satisfaction with career or that research capacity-building has a positive impact on healthcare workers’ satisfaction with their career. Right now, the paper states that there is a shortage, that practicing in rural areas is perceived as career-limiting and that the solution could be building research capacity but there is no evidence of why that would help. Why do you think this will have a strong impact on rural workforce recruitment and retention? Further, please explicitly make a connection of how building research capacity can help one’s career, especially when remaining in a rural area.

**Response**: Dear reviewer, thank you for this important comment. Evidence from Australian RCB initiatives demonstrates that engagement in structured research programs can positively influence health professionals’ career satisfaction, motivation, and retention, particularly in rural and remote settings. Participation in programs such as the RRCBP and STaRR has been associated with enhanced research skills, confidence, and employability, as well as opportunities to contribute to meaningful, locally relevant research projects (Alston et al., 2025; Flenady et al., 2022; King et al., 2025; King et al., 2023; Schmidt et al., 2022). These factors are known to increase professional fulfilment, a sense of purpose, and perceived career progression, which are critical for retaining staff in rural areas where opportunities for advancement are often limited.

By embedding research opportunities within rural workplaces, health professionals can actively participate in generating evidence to improve service delivery, strengthen local health systems, and develop leadership and mentoring skills. This not only enhances individual competence and career satisfaction but also fosters organisational research culture and social capital. These outcomes support retention by providing tangible incentives, beyond financial rewards, for health workers to remain in rural positions.

Comment 8: Line 137: Add “s” to “health professionals”

**Response**: We have revised it.

**Reviewer #2**

Comment 1: The research question outlined is expected to address a valid academic problem or topic and contribute to the base of knowledge in the field. The authors have been very restrictive in the definition of their research question and while the outcome of retention is key to the healthcare workforce (and there is a wide literature in that area), it is unclear whether it is appropriate to just look at one type of retention enhancement focused on research (and research building capability) or whether its value needs to be considered relative to other retention strategies.

**Response**: Dear reviewer, thank you for this thoughtful comment. We acknowledge the concern about focusing narrowly on research engagement and RCB when many other strategies exist to support recruitment and retention in rural and remote health settings. Our intention in this scoping review is not to imply that RCB is the only or primary retention strategy, but rather to explore an area where evidence is emerging yet remains poorly synthesised. Research engagement and RCB have been increasingly promoted as mechanisms to enhance professional identity, career satisfaction, capability, and organisational commitment, all factors known to influence attraction and retention. However, unlike other well-established retention strategies (e.g., financial incentives, training pathways, housing support), the specific contribution of RCB has not been comprehensively mapped.

To address this gap, our scoping review will examine the breadth of available evidence on how research engagement and RCB may contribute to health workforce outcomes in rural and remote Australia. The review will address four interrelated questions:

1. What evidence exists on the relationship between health workforce engagement in research and/or RCB and workforce attraction, retention, and stability in rural and remote Australia?
2. What enablers support the implementation and impact of RCB on workforce outcomes?
3. What barriers influence the effectiveness of RCB initiatives?
4. What strategies have been identified to optimise future RCB implementation to strengthen attraction, retention, and stability of the rural and remote health workforce?

By mapping this evidence, our review aims to clarify where RCB sits relative to other retention strategies and to determine whether, and in what ways, strengthening research engagement may support broader workforce sustainability efforts. This approach will also highlight gaps where future empirical research is needed.

Comment 2: Furthermore, given the lack of extensive evidence in the area of using research to enhance retention, it is unclear why the authors have chosen to focus narrowly on Australia in this project, particularly when retention issues are a global problem and there may be useful studies from other countries.

**Response**: Dear reviewer, thank you for this comment. While health workforce retention is indeed a global challenge, there remains a clear synthesised evidence gap in the Australian context regarding the impact of RCB on workforce attraction, retention, and stability. Focusing on Australia allows us to map the available evidence, identify context-specific barriers and enablers, and generate actionable insights tailored to the unique organisational, cultural, and policy environment of rural and remote Australian health services.

For future work, the scope could be expanded to include other high-income countries with comparable rural health workforce challenges, such as Canada, the United States, Brazil, or the United Kingdom. Despite its national focus, the findings from this review are likely transferable to similar settings where workforce shortages and retention difficulties exist, providing valuable guidance for designing RCB initiatives that strengthen rural and remote health workforces internationally.

Comment 3: It is unclear to me how many articles could be found to address the rather narrow research question as currently addressed in this paper. In my attempt to search on this topic for one of the databases mentioned, I came up with only 200 articles and most proved irrelevant (wrong location, wrong subject). It may be helpful for the authors to indicate what search terms will be used to capture sufficient articles on this topic.

**Response**: We thank the reviewer for this thoughtful comment and for undertaking an independent search to assess the scope of available literature. We acknowledge that the evidence base in this area is relatively limited, given the specificity of our focus on research engagement and research-capability building within the rural and remote Australian health workforce. Our preliminary searches using broad conceptual terms identified approximately 300 to 350 records in MEDLINE alone (**see Supplementary Table at the end of this document**), consistent with the reviewer’s experience. This volume is expected for a geographically and thematically focused topic.

In line with best practice for scoping reviews, our search strategy has been intentionally designed to be iterative. We began with a broad set of terms to maximise sensitivity and ensure that all potentially relevant literature, including studies that may describe research activity, capability-building, or workforce outcomes indirectly, is captured. As searches progress, we will refine and adapt our terms based on emerging patterns in the literature, database-specific indexing, and consultation with an experienced health research librarian. This iterative approach will help balance sensitivity and specificity, ensuring that no substantial body of relevant work is overlooked.

Furthermore, we will supplement database searches with a comprehensive grey literature search across Australian organisational websites, government reports, program evaluations, and other publicly available sources. Because much research-capability initiatives and workforce programs in rural Australia are not formally published in peer-reviewed journals, grey literature is essential for capturing the full breadth of evidence.

Taken together, we are confident that our approach will identify a sufficiently rich body of literature to address the review objectives while maintaining methodological rigour and transparency.

Comment 4: It is unclear why a systematic review is needed at this time when there is little evidence on the existing scope of literature in this area. It would be helpful to hear whether the authors considered a scoping review first before choosing a systematic review. Or could they explain further why the systematic review is more useful than a scoping review here. It seems unlikely that "the relationship between health workforce engagement in research or research capability-building programs and the attraction and retention of health professionals in regional Australia" can be easily compared across different studies, if, as I would surmise, existing literature in this area provides exploratory rather than confirmatory evidence.

**Response**: Dear reviewer, thank you for this constructive observation. We agree with your interpretation and confirm that our study is designed as a scoping review, not a systematic review. A scoping review is the most appropriate methodological approach at this stage because the evidence base on RCB as mechanisms to influence health workforce outcomes in rural and remote Australia remains limited, conceptually diverse, and largely exploratory rather than evaluative. Our objective is to map the extent and nature of the existing literature, examine how RCB has been defined and operationalised, and identify the key enablers, barriers, and strategic approaches reported across studies. This approach will also allow us to highlight evidence gaps and consider insights from comparable international settings, particularly high-income countries with similar rural and remote workforce challenges.

By first conducting a scoping review, we can establish a comprehensive foundation that will guide future, more focused systematic reviews or meta-analyses when a sufficiently mature and methodologically comparable evidence base becomes available.

Comment 5: Overall, the authors have an interesting idea to identify ways that research and research-enhancing work supports a remote workforce, but their questions are too narrowly defined and they could learn more by comparing across countries.

**Response**: Thank you for this helpful comment. We recognise the potential value of international comparisons; however, our scoping review is limited to the Australian context for several reasons. First, rural and remote health systems in Australia operate within a distinctive policy, governance, and service delivery environment. RCB initiatives are shaped by Australian funding structures, workforce models, and priorities related to Aboriginal and Torres Strait Islander health, factors that differ substantially from other countries and limit the direct applicability of international evidence.

Second, there is a clear gap in the Australian literature regarding how RCB contributes to workforce attraction, retention, and stability in rural and remote areas. Australia has a number of locally developed RCB programs, such as the RRCBP and STaRR, that have not yet been synthesised in a focused review. Restricting the scope to Australian studies allows us to examine these programs in depth and respond to a specific national evidence need.

Finally, our review is designed to support decision-making for Australian health services and policymakers. A country-specific synthesis ensures that findings are directly relevant to national workforce challenges and can guide future investments in research development within rural and remote settings.

For these reasons, we have chosen to focus exclusively on the Australian context while acknowledging that comparisons with similar countries may be a useful direction for future work.

**References**

Alston, L., Pu, D., Wood, S., Field, M., Beks, H., King, O. A., Wheaton, N., Huggins, C. E., Shee, A. W., Namara, K. M., & Versace, V. L. (2025). Embedding research into the organisational structure of smaller rural hospitals: building research culture and capacity and understanding perceived rural health workforce benefit. *BMC Health Services Research*, *25*(1), 1008. <https://doi.org/10.1186/s12913-025-13107-8>

Cooke, J. (2005). A framework to evaluate research capacity building in health care. *BMC Fam Pract*, *6*, 44. <https://doi.org/10.1186/1471-2296-6-44>

Dako-Gyeke, P., Asampong, E., Afari, E., Launois, P., Ackumey, M., Opoku-Mensah, K., Dery, S., Akweongo, P., Nonvignon, J., & Aikins, M. (2020). Capacity building for implementation research: a methodology for advancing health research and practice. *Health Research Policy and Systems*, *18*(1), 53. <https://doi.org/10.1186/s12961-020-00568-y>

Flenady, T., Dwyer, T., Kahl, J., Sobolewska, A., Reid-Searl, K., & Signal, T. (2022). Research capacity-building for clinicians: understanding how the research facilitator role fosters clinicians’ engagement in the research process. *Health Research Policy and Systems*, *20*(1), 45. <https://doi.org/10.1186/s12961-022-00849-8>

King, O. A., Beks, H., Beauchamp, A., Namara, K. M., Sayner, A. M., West, E., & Shee, A. W. (2025). Implementation and development of an embedded researcher program driven by rural health services: a qualitative exploratory case study. *BMC Health Services Research*, *25*(1), 1198. <https://doi.org/10.1186/s12913-025-13336-x>

King, O. A., Sayner, A. M., Beauchamp, A., West, E., Aras, D., Hitch, D., & Wong Shee, A. (2023). Research translation mentoring for emerging clinician researchers in rural and regional health settings: a qualitative study. *BMC Medical Education*, *23*(1), 817. <https://doi.org/10.1186/s12909-023-04786-0>

Matus, J., Walker, A., & Mickan, S. (2018). Research capacity building frameworks for allied health professionals – a systematic review. *BMC Health Services Research*, *18*(1), 716. <https://doi.org/10.1186/s12913-018-3518-7>

Schmidt, D., Duncanson, K., Webster, E., Saurman, E., & Lyle, D. (2022). Critical realist exploration of long-term outcomes, impacts and skill development from an Australian Rural Research Capacity Building Programme: a qualitative study. *BMJ open*, *12*(12), e065972. <https://doi.org/10.1136/bmjopen-2022-065972>

**Supplementary Table.** Search results from **MEDLINE** database using the keywords and subject heading across the four concept.

| **#** | **Search strategy** | **Results** |
| --- | --- | --- |
| 1 | ((("health care" or healthcare or health or medical or nurse) adj2 (personnel or professional* or provider* or staff or worker* or manpower)) or ("human resource*" or Internist or doctors or "allied health" or audiologist or "dental staff" or dentist or dietician) or ((language or speech) adj2 (pathologist or therapist)) or (midwi* or nurse or nutritionist or "occupational therapist*" or ophthalmologist* or p?diatrician or pharmacist* or physician or "general practitioners" or surgeon or radiologist or radiographer or psychologist or physiotherapist or "physical therapist")).ti,ab. | 1,141,931 |
| 2 | exp Health Personnel/ or exp Workforce/ or exp Health Workforce/ or exp Physicians/ or exp Allied Health Personnel/ or exp Allied Health Occupations/ or exp Nurses/ or exp Health Occupations/ | 2,470,093 |
| 3 | #1 OR #2 | 3,191,912 |
| 4 | (((Capability or Capacity) adj2 (building or development)) or (Research adj2 (activities or collaboration or engagement or initiatives or involvement or mentorship or network or participation or partnership or project or programs or skills or support or training)) or ((action or participatory) adj research) or ("analytical skills" or "programme evaluation") or (knowledge adj2 ("utilisation research" or building or transfer)) or (scientific adj2 (evidence or knowledge or Skill))).ti,ab. | 139,250 |
| 5 | exp Capacity Building/ or exp Mentoring/ or exp Mentors/ or exp Research/ or exp Health Services Research/ or exp Program Evaluation/ or exp Evidence-Based Practice/ or exp Evidence-Based Medicine/ or exp Translational Research, Biomedical/ or exp Nursing Research/ | 1,045,276 |
| 6 | #4 OR #5 | 1,148,888 |
| 7 | (((employee or employment or staff or talent) adj2 (attraction or benefits or engagement or loyalty or reward or retain or retention or recruitment or turnover or well-being or "work-life balance")) or ((organisational or organizational) adj2 (change or characteristic* or culture or determination or innovation or process or support or commitment or impact or outcome)) or ((workforce or work or job) adj2 (development or engagement or influence or motivation or satisfaction or security or stability)) or "intention to leave").ti,ab. | 50,099 |
| 8 | Job Satisfaction/ or Personnel Management/ or Personnel Turnover/ or exp Organizational Innovation/ or Organizational Culture/ or exp Staff Development/ or exp Burnout, Professional/ or exp Job Security/ or exp Efficiency, Organizational/ or exp Workplace/ or exp employment/ | 227,368 |
| 9 | #7 OR #8 | 256,999 |
| 10 | (Australia* or Canberra or "new south Wales" or Sydney or Victoria or Melbourne or Queensland or Brisbane or Adelaide or Tasmania or Hobart or Perth or "western Australia " or Darwin or "northern territory").ti,ab. | 229,520 |
| 11 | exp Australia/ or exp Australian capital territory/ or exp new south Wales/ or exp northern territory/ or exp Queensland/ or exp south Australia/ or exp Tasmania/ or exp Victoria/ or exp western Australia/ | 186,477 |
| 12 | #10 OR #11 | 281,792 |
| 13 | (((Country or countryside or nonmetropolitan) adj2 (healthcare or "health care" or "health services" or "health facilities" or "medical service" or "health sector")) or ((deprived or disadvantaged) adj2 (area or community or groups or province or region)) or ((remote or rural) adj2 (area or clinic or community or health or "health care" or healthcare or "health service*" or hospitals or "medical care" or "medical services" or province or region or settings or population)) or "shortage area" or ((unequal or inequitable) adj distribution)).ti,ab. | 63101 |
| 14 | exp Rural Health/ or exp Rural Health Services/ or exp Primary Health Care/ or exp Vulnerable Populations/ or exp Medically Underserved Area/ or exp Healthcare Disparities/ | 288,496 |
| 15 | #13 OR #14 | 337,403 |
| 16 | #3 AND #6 AND #9 AND #12 AND #15 | 321 |
